# A Generalizability Analysis of the Meaning in Life Questionnaire for Chinese Adolescents

**DOI:** 10.3389/fpsyg.2021.687589

**Published:** 2021-11-25

**Authors:** Wei Chen, Rongfen Gao

**Affiliations:** ^1^School of Psychology, Guizhou Normal University, Guiyang, China; ^2^Center for Big Data Research in Psychology, Guizhou Normal University, Guiyang, China

**Keywords:** generalizability theory, Meaning in Life Questionnaire, MLQ, Chinese adolescents, generalizability coefficient, generalizability analysis

## Abstract

The level of meaning in life not only affects the physical health of individuals, but also is closely related to their mental health. At present, many self-reported questionnaires are being used to measure the meaning in life of Chinese adolescents. Using the multivariate generalizability theory, this study investigated the psychometric properties and the internal structure of the Meaning in Life Questionnaires (MLQs), the most widely used questionnaire for assessing the level of meaning in life of Chinese adolescents. The data were sample of 1,951 junior high school students from Guizhou, China. Multivariate random measurement mode *p* × *i*° is the primary analytic approach. Results showed that the generalizability coefficient and dependability index of the scale were 0.86 and 0.85, respectively. The generalizability coefficients of presence of meaning and search for meaning were 0.76 and 0.85, respectively, and the dependability indexes were 0.75 and 0.85 for MLQ-P and MLQ-S, respectively. The design of each factor for MLQ is reasonable in terms of score ratio and the number of projects. In brief, the reliability and factor structure of the scale are satisfactory.

## Introduction

Adolescence is accompanied by significant changes in the decision-making process of meaning, values, and goals. Finding the meaning in life and establishing a coherent philosophy of life have become the key issues at this stage (Krok, 2018). The level of meaning in life not only affects the mental health of individuals but also is closely related to their physical health (Brassai et al., 2011). It will have a far-reaching influence on the development of individuals, who try to explain and organize their experiences by identifying important aspects of their personal and social life and find deeper meaning in their lives when encounter new situations and events (Krok, 2018).

Accessible studies showed that the meaning in life is positively associated with positive affect, emotions, subjective and psychological well-being (Krok, 2018), mental health (Miao and Gan, 2020), and psychological and academic adjustment (Kiang and Fuligni, 2010) and negatively associated with hopelessness, negative focus, suicide (Lew et al., 2020), and bad behavior and habits among adolescents (e.g., illicit drug and sedative use, unsafe sex, binge drinking, and lack of exercise and diet control) (Brassai et al., 2011). The meaning in life is an important protective factor of suicide (Lew et al., 2020), health risk behaviors and poor psychological health (Brassai et al., 2011), and post-traumatic stress disorder (PTSD) (Bryan et al., 2020). The meaning in life is considered one of the conditions related to growth (Steger et al., 2006) and is a valuable evaluation index of positive psychological function (Krok, 2018).

The meaning in life is defined as “the sense made of, and significance felt regarding, the nature of one’s being and existence” (Steger et al., 2006). It includes two aspects, namely, presence of meaning and search for meaning. The presence of meaning refers to the degree of individual feeling about whether they live a meaningful life, while search for meaning refers to the degree of active search of an individual for meaning in life (Steger et al., 2006). The former emphasizes the result of feeling the meaning in life, while the latter emphasizes the process of finding the meaning in life. Previous studies indicated that the presence of meaning and search for meaning may promote the generation of prosocial behavior (Wang et al., 2018). Additionally, the results revealed that the higher presence of meaning was associated with lower health anxiety, while the relationship between the search for meaning and health anxiety was opposite (Yek et al., 2017). A meta-analysis study concluded that the presence of meaning is closely related to higher subjective wellbeing. In general, the search for meaning has less impact on subjective wellbeing, and it is conditional to a certain extent (Li et al., 2021). Therefore, the significance of presence of meaning and search for meaning to individual psychology is not completely consistent, i.e., the two factors are essentially different.

Many scholars had developed measurement instruments to evaluate the sense of meaning in life of an individual, such as the Purpose in Life Test (PIL) (Crumbaugh and Maholick, 1964), the Life Regard Index (LRI) (Battista and Almond, 1973), and the Life Attitude Profile – Revised (LAP-R) (Reker, 1992). However, these research instruments were questioned due to the problem of factor structure or content validity (Steger et al., 2006; Brandstatter et al., 2012). To make up for these deficiencies, Steger et al. (2006) developed Meaning in Life Questionnaire (MLQ). It is composed of two factors, which were used to measure the presence of meaning (MLQ-P) and search for meaning (MLQ-S), respectively, and had good reliability, where both the internal consistency coefficient and the retest coefficient are greater than 0.70 (Steger et al., 2006). It is favored by many scholars because of its good psychometric performance and simplicity and is used to study the meaning in life in different cultural backgrounds.

The MLQ has been translated into various versions, and the factor structure of the scale is supported by empirical studies carried out in countries such as India (Negri et al., 2020), Australia (Rose et al., 2017), Greece (Pezirkianidis et al., 2016), Brazil (Damásio et al., 2015), South Africa (Temane et al., 2014), and Turkey (Boyraz et al., 2013). At the same time, since the development of the scale, a few studies have been performed on the investigation of the measurement quality of MLQ among Chinese adolescents (Wang, 2013; Chen et al., 2017). The results of these studies reported that the alpha coefficients of the total scale were 0.736 and 0.830, respectively, and the alpha coefficients of the two subscales ranged from 0.649 to 0.842. In addition, the test-retest reliability of the total scale was 0.639, the test-retest reliability of the two subscales was between 0.558 and 0.746 (Wang, 2013), and the fitting indicators were good (Wang, 2013; Chen et al., 2017).

However, it is worth noting that all the studies mentioned earlier are based on the Classical Test Theory (CTT) to verify the psychometrical properties of MLQ. The parameters used to evaluate the scale (e.g., reliability, validity, difficulty, and discrimination) obtained through methods under the CTT have been heavily dependent on the adolescents selected in each empirical study. Therefore, the results of these analyses can only evaluate current research, and the information present in the questionnaire should not be promoted. The reason is that when CTT is used to examine the reliability and validity of a test, there are some limitations, such as the estimation of test reliability is not accurate enough; it cannot distinguish the various sources of variation and their magnitude that may occur during the test; and it is unable to propose strategies and plans to reduce measurement errors (Yang and Zhang, 2003; Suen and Lei, 2007).

Therefore, many researchers have been looking for ways to overcome these shortcomings. One of the research directions is to start from the external or macro aspects of the test, continue to develop along the idea of random sample theory, and investigate the measurement conditions and conclusions of the test preparation. The relationship between the scope of application is to focus on the external validity of the test. Along this line of thought, researchers created and developed the generalizability theory (GT) of measurement (Cronbach et al., 1963). Compared with CTT, GT can divide the total error into multiple component errors according to the source of measurement error and perform reliability analysis based on considering multiple sources of error at the same time (Nußbaum, 1984). Therefore, the reliability analysis of GT is more detailed and accurate than CTT.

The types of GT include univariate generalization theory (UGT) method and multivariate generalization theory (MGT) method. Among the GT types, the advantages of MGT are more obvious, especially in the reliability and validity evaluation of multidimensional measurement tools. It can estimate not only the variance component (VC), generalization coefficients, and dependent indicators of the total score of the scale but also the VCs, covariance component, and generalizability of each dimension. The internal structure of the scale can be analyzed in depth through the covariance components of different dimensions, that is, the rationality of the size of scale (Nußbaum, 1984; Clauser et al., 2002, 2006; Yin, 2005).

Generalizability theory contains two stages, namely, Generalizability Studies (G Study) and Decision Studies (D Study). The main purpose of G study is to clarify the test design, including the analysis of the measurement objectives, test structure, measurement objects, and measurement modes and items. This method uses the variance analysis technology to decompose the variation of the test total score, in order to clarify the relationship between various factors and estimate the variance and covariance component matrix of each effect on each potential factor. The main purpose of D study is to explore the generalizability coefficient and reliability index, as well as the change relationship between them and measurement target or various secondary factors, so as to accurately estimate the reliability of measurement results under various decisions, and provide a necessary basis for improving measurement design and measurement quality. The content involved is to estimate the overall score of the participants on each potential factor, as well as the corresponding relative error, absolute error, generalizability coefficients and reliability indexes, and then determine the weight coefficient of the global scores of each factor by using methods such as covariance contribution rate, so as to synthesize a global total score (Composite Universe Score), and estimate its corresponding relative error, absolute error, generalizability coefficient, reliability index, relative signal-to-noise ratio and absolute signal-to-noise ratio. The final step is to make corresponding decisions based on the estimated results (Nußbaum, 1984; Yang and Zhang, 2003).

In addition, based on the combined relationship between the measurement object and the measurement facet in GT, it is divided into three measurement modes, namely, random measurement mode, fixed measurement mode, and mixed measurement mode. The random measurement mode refers to a conditional sampling in which the measurement facet is randomly selected from the acceptable observation range; in this context, the measurement mode is random measurement mode and the aspect is random. In contrast, the fixed measurement mode (i.e., standardized test in CTT) refers to the context in which the conditional sampling of the measurement facet is fixed, and the facet is also a fixed facet. However, the mixed measurement mode indicates that both random and fixed facets are present in the measurement process (Yang and Zhang, 2003). In the light of the measurement instruments of the meaning in life developed by previous researchers, it can be seen that the measurement dimensions of different scales are not the same, and it can be considered that they are randomly selected from the universe where the meaning in life dimension can be observed. Hence, this study has chosen the random measurement mode.

This study would use MGT to evaluate the psychometric properties of MLQ and examine the internal structure of the two dimensions of the scale. This study not only helps to improve the measurement accuracy and structure of the scale but also provides a reference for the practical application of the scale. This study is expected to answer the following hypotheses:

H1: The variance component of the two factors in the subject effect and the interaction between the subject and the project would be large, but the item effect would be small.

H2: The absolute error and relative error of the universe score of scale would be small, while the generalization coefficient and dependent index of the total score would be large.

## Materials and Methods

### Participant

Through the method of facilitating cluster sampling, 2,000 middle school students were randomly selected from Guizhou, China. The inter-class time of students were used to conduct paper-and-pencil tests on students who volunteered to participate in this study. Recovered 1,951 valid questionnaires, including 1,015 (52.4%) females and 923 (47.6%) males, and 13 participants were tried not to complete the gender fill in a column, which is encoded as missing. In addition to 12 participants who were tried not to report their age, the rest of them were 12–18 years old (*M* + *SD* = 13.47 + 1.306).

### Procedure

Before the study, we obtained the consent from parents and teachers of the students, explained the purpose of data collection to students, and strictly abided by the principles of confidentiality and voluntary participation. Before the survey, we also provided the participants with the paper informed consent form, which was distributed to all participants together with the paper questionnaire. The formal questionnaire survey was not started until the students filled in the informed consent form. After the survey, the investigators will take back the questionnaire uniformly. The whole process took about 15 min. This study was approved by Committee of the School of Psychology of Guizhou Normal University.

### Assessments

The level of meaning in life was measured by using MLQ (Steger et al., 2006), which is a multidimensional self-report instrument. It consists of two dimensions, namely, MLQ Presence (MLQ-P) and Search (MLQ-S), each of them containing five items. The former is used for the degree to which individuals perceive the meaning of their own life (e.g., “My life has a clear sense of purpose”), while the latter is used to evaluate the degree to which individuals need the meaning in life (e.g., “I am always searching for something that makes my life feel significant”). Items were scored on a 7-point scale (1 = *absolutely untrue*, 7 = *absolutely true*). The MLQ had a good internal consistency (α = 0.819; the alpha coefficients of MLQ-P and MLQ-S were 0.759 and 0.848, respectively).

### Measurement Design

The one-facet multivariate design *p* × *i*° in the MGT was used to analyze the MLQ data among adolescents. This is a traditional form with a standardized design with fixed content categories. Each item is nested in one and only one content category (Brennan, 2001b). Participant (*p*) is the measurement object and items (*i*) of each dimension are the measurement facets. In addition, where the superscript filled circle designates that persons are the same across categories, and the superscript empty circle designates that items are different across categories (Brennan, 2001a, b). They are assumed to be completely random and have a cross relationship. The generalizability design used mGENOVA software packages for data processing and statistical analyses (Brennan, 2001a; Yang and Zhang, 2003).

## Results

### Descriptive

The minimums, maximums, means, standard deviations, and zero correlations among the two dimensions of the MLQ are presented in Table 1.

**TABLE 1:** Minimums, maximums, means, standard deviations (SD), and bivariate correlations among the four dimensions.

**Variable**	**Descriptive statistics**					**Correlations**	
	***N***	***Min***	***Max***	***Mean***	***SD***	**MLQ-P**	**MLQ-S**
MLQ-P	1951	5	35	21.36	6.686	1	
MLQ-S	1951	5	35	23.55	6.611	0.357***	1

MLQ-P, presence of meaning; MLQ-S, search for meaning. ***Correlation is significant at the 0.001 level.

### Results of MGT G Study

According to the research design of the two-factor dimension model, the estimation matrix of the variance and covariance component of the participant (*p*), item (*i*), and the interaction between the participant and the item (*pi*) can be obtained on all two-factor dimensions (presence of meaning and search for meaning) in the G study stage. Results are shown in Table 2. The VCs of the items in each dimension were less than 0.11546, showing that the items had no significant impact on the total variation of the scale. In terms of the two dimensions, the covariance components of the presence of meaning and search for meaning among the effects of participants were relatively large (0.62174). The correlation coefficient among the dimensions was 0.44502. In summary, the covariance components between the two factors are relatively high, which is a basis for factor scores to synthesize the total score. Whether it can be synthesized, we should further refer to the results of the D study. In addition, the VCs of the two dimensions in the interaction effects between the participants and the items were relatively large (2.09094 and 1.33026), indicating that the interaction effects between the participants and the items had a greater impact on the total variation of the scale. However, whether the test can be used as a normative reference test or a standard reference test requires further reference to the results of the D study.

**TABLE 2:** Estimated variance and covariance components for *p* × *i*° design in MGT G study for the two dimensions of the MLQ (*N* = 1,951).

	**MLQ-P**	**MLQ-S**
*Participant (p)*	**1.31676**	0.44502
	0.62174	**1.48232**
*Items (i)*	**0.11546**	
		**0.00762**
*Participant by items (pi)*	**2.09094**	
		**1.33026**

*MGT, multivariate generalizability theory. Diagonal elements are estimated variance components and are presented in bold type. The lower diagonal elements are covariances, and the upper diagonal elements are correlations.*

### Results of MGT D Study

According to the variance and covariance matrix estimated by G study, the universe score of the two factors and the VC of the corresponding error can be estimated in the D study stage and then the estimated value of the generalized coefficient, the reliability index, the relative signal-to-noise ratio, and the absolute signal-to-noise ratio were obtained. Results were displayed in Table 3. The generalizability coefficient (0.85524) and the dependent index (0.85081) of the universe score of scale were large, even larger than that for MLQ-P and MLQ-S. In contrast, the relative error variance (0.17106) and the absolute error variance (0.17721) of the universe score of scale were significantly lower than the error variance of each dimension. The generalizability coefficient and dependent index of MLQ-S were all greater than 0.84. Additionally, the generalizability coefficient and dependent index of MLQ-P were greater than 0.74. All in all, the overall scale and subdimensions have reached a very ideal level.

**TABLE 3:** Estimated MGT D study statistics for the MLQ.

**Index**	**MLQ-P**	**MLQ-S**	**Universe score**
USV	1.31676	1.48232	1.01064
REV	0.41819	0.26605	0.17106
AEV	0.44128	0.26758	0.17721
EVM	0.02398	0.00242	0.00676
GC	0.75896	0.84783	0.85524
DI	0.74899	0.84709	0.85081
S/NR	3.14873	5.57155	5.90810
S/NA	2.98395	5.53981	5.70292

*USV, universe score variance; REV, relative error variance; AEV, absolute error variance; EVM, error variance for mean; GC, generalizability coefficient; DI, dependent index; S/NR, signal/noise relative; S/NA, signal/noise absolute.*

### Contribution Ratio of Each Dimension

The weight coefficient (ω) is based on the percentage of the number of items in each factor to the total items. As shown in Table 4, the weight coefficients of both MLQ-P and MLQ-S were 0.50. The contribution of MLQ-S to the universe score variance is higher than their contribution to the total score, while the contribution of MLQ-P to the universe score variance is slightly lower than its contribution to the total score. Thus, MLQ-P contributes more to relative error variance and absolute error variance than MLQ-S.

**TABLE 4:** Contribution ratio of each dimension to the universe score of scale.

**Index**	**MLQ-P**	**MLQ-S**
Number of dimension	5	5
Total score of each dimension	35	35
ω	0.500	0.500
Score ratio the for each dimension	50%	50%
Contributions to the universe score variance	47.95%	52.05%
Contributions to the relative error variance	61.12%	38.88%
Contributions to the absolute error variance	62.25%	37.75%

## Discussion

In this study, MGT was used to further explore the reliability and validity of MLQ among adolescents and to make up for the rough side of the estimation of CTT for the reliability and validity of MLQ. The results found that the variance components of each factor of MLQ in the effect of participants were relatively high; thus, H1 has been verified. Also, there was no obvious difference indicating that the participants and the variation related to the participants accounted for a large proportion of the total variation of the test. In addition, the covariance components of each factor are not much different, and the correlation coefficient is low, showing that the factors are both related and independent and that the two-factor theory of meaning in life is suitable for Chinese adolescents, thus verifying the previous research conclusions again (Wang, 2013). In contrast, in terms of the effects of items, the proportion of VCs of each factor is small, indicating that the proportion of variance caused by the item is small and that the quality of the MLQ items is satisfactory (with good difficulty and discrimination), which are consistent with the findings of previous scholars (Chen et al., 2017). Most importantly, we can also draw a conclusion that these items are not the main source of measurement error through generalization analysis (Nußbaum, 1984), which can be used to measure the level of meaning in life of adolescent and effectively distinguish the differences between individuals.

In terms of the two-factor structure, if the two factors are combined into the total scale, the generalization coefficient and reliability index of the overall scale reach more than 0.85, with good reliability; thus, H2 has been verified. In addition, the relative error variance and absolute error variance of the overall scale are obviously lower than each factor, indicating that it is feasible and effective to use the combined total table of the two factors. The generalization coefficient and reliability index of each factor are greater than 0.74, reaching the satisfactory level, indicating that it is reasonable to divide MLQ into two factors, and the measurement superiority of the scale is good. Consistent with the previous research results, the factor structure is reasonable (Steger et al., 2006; Wang, 2013; Chen et al., 2017). Finally, through the analysis of variance contribution rate, it is found that the contribution of the two factors to the total global score is significantly different from their proportion in the total score table, which may be caused by the reverse scoring in MLQ-P factor.

To sum up, both the overall scale and each factor as a single scale can be used as norm reference test and standard reference test in the adolescent group to measure the level of individual life meaning. However, as to whether it is a relative decision or an absolute decision, the researcher needs to consider the purpose of use. If used to distinguish the level of meaning in life in adolescents (norm reference test), relativistic interpretation of the measurement results is required. When used to assess the true level of a subject (standard or target reference test), absolute interpretation of the measurement results is required. They all need to refer to the generalization coefficient and reliability index (usually > 0.8) to make the corresponding decision. In terms of the two-factor structure, the overall scale and all factors are satisfied, so two explanations can be given.

Although our research has obtained meaningful results and enriched the research theory and methodology of MLQ, there are also limitations. First, the participants of this study are mainly from Guizhou Province, China; in the future research, it is necessary to combine more resources to expand the scope of research. Moreover, the current research participant is mainly ordinary young students, which has not been involved in the special group. In further research, we could comprehensively consider the applicability of MLQ in different groups (e.g., Special youth). Most significantly, when carrying out mental health education activities, especially for some individuals who are facing great disasters and emergencies, it is necessary for us to evaluate the level of meaning in life, especially to pay attention to the presence of meaning and search for meaning. Specifically, in face of individuals with a high presence of meaning in life, to a certain extent, we can confirm that their mental health level is relatively stable. On the contrary, if the level of meaning in life is low and search for meaning is not high, we should pay more attention to this group.

## Conclusion

This study can draw the following two conclusions: (1) the MLQ scale has high reliability and validity, which can be used not only as a norm reference test but also as a standard reference test and (2) the score ratio of each factor of MLQ and the design of the number of items are reasonable to perfect.

## Data Availability Statement

The original contributions presented in the study are included in the article/Supplementary Material, further inquiries can be directed to the corresponding author.

## Ethics Statement

The studies involving human participants were reviewed and approved by the Ethics Committee of the School of Psychology, Guizhou Normal University. Written informed consent to participate in this study was provided by the participants’ legal guardian/next of kin.

## Author Contributions

WC concepted the manuscript, provided framework of the manuscript, and approved final version of the manuscript. RG analyzed the data and drafted the manuscript. Both authors contributed to the article and approved the submitted version.

## Conflict of Interest

The authors declare that the research was conducted in the absence of any commercial or financial relationships that could be construed as a potential conflict of interest.

## Publisher’s Note

All claims expressed in this article are solely those of the authors and do not necessarily represent those of their affiliated organizations, or those of the publisher, the editors and the reviewers. Any product that may be evaluated in this article, or claim that may be made by its manufacturer, is not guaranteed or endorsed by the publisher.
